# Supplementary material for: Multilevel analysis of unhealthy bodyweight among women in Malawi: Does urbanisation matter?
Source: PLoS One. 2021 Mar 29;16(3):e0249289. doi: 10.1371/journal.pone.0249289 (PMC8006991; doi:10.1371/journal.pone.0249289)
Supplement: Attachment — Submitted filename: PLOS one Review.docx [file pone.0249289.s001.docx]

The authors have used multi-level multinomial logistic regression analysis to assess the net effects of urban residence on the double burden of malnutrition amidst individual and community level factors among women in Malawi.

Abstract

The authors indicate unhealthy weight, underweight and overweight as symptomatic of the dual burden of DBM. Stating, unhealthy weight, then underweight and overweight at the same time would imply that overweight and underweight are different from unhealthy weight whereas they are both unhealthy weight. I would suggest correction of this sentence. The sentence does not read well.

Under the results in the abstract and the entire paper you use the phrase, 'INSIGNIFICANT' i am not sure if this is the right word particularly when referring to a statistical outcome. I am wondering if 'non-significant' will make the interpretation sound better. Explore which is the best word to use and correct accordingly. You also indicate that ethnicity was a significant risk factor of DBM, can you specify which ethnic group and perhaps put the aRR and CIs. This will help the reader to make a follow-up in the paper, why this particular ethnic group (s) is peculiar. Line 40 please replace the word likely with the word likelihood and insert while before breast feeding.

Background

This section is well written and provides the background, country context and why it is necessary to study net effects of urban residence on DBM in Malawi.

Methods section

This section is also explicitly written. I am not sure about the link between the explanation of the study, objective and the outcome variable. In the title you are talking about unhealthy body weight, in the objective DBM while the outcome variable is BMI. The link between this three is necessary. Although all these concepts are related they do not mean the same thing and there is need for authors to carefully provide consistency in what the study really seeks to address. I don't think expressing the MMLR model in formula is necessary for the reader.I would rather have it as an attachment separate from the main manuscript and describe briefly how models were constructed at the time of data analysis.

Results

The results section is well written/ The conventional way to indicate statistical significance at 95% CI would be *** instead of *. Under the Adjusted model's random effects-line 257, you used abbreviations such as ICC, PCV and AIC values without first explaining their meaning.

Discussion

this section is also generally well written. However, line 316-318 is not necessary because you have earlier stated the objected of the study. In line 342 you indicate that ever married women were at risk of being overweight while un-married were at risk of underweight.In justifying why married women are more overweight you posit that it is because married women are exposed to pregnancy and child birth which lead to hormonal and physiological body-weight changes.One interesting question would be that, are un-married women not exposed to these body changes, or it is that there are low levels of pregnancies among unmarried women in Malawi??

In line 364 -367, the discussion is not relevant because you indicate that after adjusting for covariates wealth status was not a significant contributor to unhealthy weight. It would be relevant if you would explain why the non-variation in unhealthy weight is observed in Malawi...You use the word Aside?? i am not sure what it means, did you mean besides?

General comments

The article with correcting minor comments is publishable.
