# Supplementary material for: Multilevel analysis of unhealthy bodyweight among women in Malawi: Does urbanisation matter?
Source: PLoS One. 2021 Mar 29;16(3):e0249289. doi: 10.1371/journal.pone.0249289 (PMC8006991; doi:10.1371/journal.pone.0249289)
Supplement: Attachment — Submitted filename: Point by point response letter_1003.docx [file pone.0249289.s002.docx]

10 March, 2021

The Editor,

PLOS|ONE,

**Cover Letter: PLOS ONE Decision: Revision required [PONE-D-20-37443] - [EMID:3d0d35eb1aa4a027]**

Thank you for the review of our paper titled “**Multilevel analysis of unhealthy bodyweight among women in Malawi: does urbanisation matter?**” The comments are valuable in terms of enriching the quality of the paper.

Please find the responses to the editorial and reviewer’s comments below for your information and attention.

Regards!

Yours sincerely,

Rotimi F. Afolabi

Mobile telephone: +234-806-607-5196

Email address: [rotimifelix@yahoo.com](mailto:rotimifelix@yahoo.com)

rotimifelixi@yahoo.com, +234 -806 607 5196

**Reviewers Notes and Responses**

| **S/N** | **Comments** | **Authors’ reply and Amendment Made** |
| --- | --- | --- |
| I | **Editorial comment - Journal Requirements** | |
| 1. | Please ensure that your manuscript meets PLOS ONE's style requirements, including those for file naming. The PLOS ONE style templates can be found at https://journals.plos.org/plosone/s/file?id=wjVg/PLOSOne_formatting_sample_main_body.pdf and https://journals.plos.org/plosone/s/file?id=ba62/PLOSOne_formatting_sample_title_authors_affiliations.pdf | We appreciate these comments. This has been addressed in the revised manuscript by replacing “Background” with “Introduction”  Please refer to the   - **Introduction, page 3, line 54** |
| II | **Reviewer #1: Comments** |  |
| 1. | The authors have used multi-level multinomial logistic regression analysis to assess the net effects of urban residence on the double burden of malnutrition amidst individual and community level factors among women in Malawi. | Thank you for this comment and recommendation. |
| 2. | Abstract   - The authors indicate unhealthy weight, underweight and overweight as symptomatic of the dual burden of DBM. Stating, unhealthy weight, then underweight and overweight at the same time would imply that overweight and underweight are different from unhealthy weight whereas they are both unhealthy weight. I would suggest correction of this sentence. The sentence does not read well. - Under the results in the abstract and the entire paper you use the phrase, 'INSIGNIFICANT' i am not sure if this is the right word particularly when referring to a statistical outcome. I am wondering if 'non-significant' will make the interpretation sound better. Explore which is the best word to use and correct accordingly. - You also indicate that ethnicity was a significant risk factor of DBM, can you specify which ethnic group and perhaps put the aRR and CIs. This will help the reader to make a follow-up in the paper, why this particular ethnic group (s) is peculiar. - Line 40 please replace the word likely with the word likelihood and insert while before breast feeding | This very important observation is highly appreciated. We have corrected the setence in the result section of the abstract accordingly.  Kindly refer to the   - **Abstract, page 2, lines 22-23**   We have replace the word “insignificant” with “non-significant” in the revised version of the manuscript.  We thank the reviewer for this observation. We have specified the **three** ethnic groups associated with both unhealthy weights. However, we do not include any of the six (3 for overweight; 3 for underweight) aRRs and CIs in order to avoid clumsiness  Please refer to  **Abstract, page 2, line 39**  Based on the comment, we have effected the changes in the revised version of the manuscript.  Kindly refer to the   - **Abstract, page 2, lines 41-42** |
| 3. | Background  This section is well written and provides the background, country context and why it is necessary to study net effects of urban residence on DBM in Malawi. | Thank you for this comment and recommendation |
| 4. | Methods section   - This section is also explicitly written. - I am not sure about the link between the explanation of the study, objective and the outcome variable. In the title you are talking about unhealthy body weight, in the objective DBM while the outcome variable is BMI. The link between this three is necessary. Although all these concepts are related they do not mean the same thing and there is need for authors to carefully provide consistency in what the study really seeks to address. - I don't think expressing the MMLR model in formula is necessary for the reader. I would rather have it as an attachment separate from the main manuscript and describe briefly how models were constructed at the time of data analysis | - We appreciate this comment - We thank the reviewer for this observation!   In line with the reviewer comment, we have revised and made necessary changes as appropriate in the revised version of the manuscript.   - We appreciate the reviewer for this important observation. However, we hope the detailed expression of the MMLR model in formula may be useful for interested reader to conduct similar “multilevel analysis” |
| 5. | Results   - The results section is well written - The conventional way to indicate statistical significance at 95% CI would be *** instead of *. - Under the Adjusted model's random effects-line 257, you used abbreviations such as ICC, PCV and AIC values without first explaining their meaning. | - This very important comment is appreciated. - We have revised and reflected the conventional way of indicating statistical significance - * p<0.05, ** p<0.01, *** p<0.001 for Tables 1 and 2 of the revised version of the manuscript.   Kindly refer to  **Results section, Tables 1 and 2** in the revised manuscript   - The abbreviations have first appeared and explained under the method section   Kindly refer to:  **Methods section, page 10, lines 214-226** |
| 6. | Discussion   - this section is also generally well written. - However, line 316-318 is not necessary because you have earlier stated the objected of the study.        - In line 342 you indicate that ever married women were at risk of being overweight while un-married were at risk of underweight.In justifying why married women are more overweight you posit that it is because married women are exposed to pregnancy and child birth which lead to hormonal and physiological body-weight changes.One interesting question would be that, are un-married women not exposed to these body changes, or it is that there are low levels of pregnancies among unmarried women in Malawi?? - In line 364 -367, the discussion is not relevant because you indicate that after adjusting for covariates wealth status was not a significant contributor to unhealthyweight. It would be relevant if you would explain why the non-variation in unhealthy weight is observed in Malawi... - You use the word Aside?? i am not sure what itmeans, did you mean besides? | - We appreciate these comments of the reviewer! - We sincerely apologise that we could not precisely understand what the comment addresses as **lines 316-318** list the significant factors peculiar to only overweight - Special thanks to the reviewer for this observation and the authors equally share the concerns of the reviewer.   Based on the observation, the statement has been modified.  Please refer to:  **Discussion section, page 20, line 358**  for the modifications provided in the revised manuscript   - We agree totally with this comment of the reviewer. However, employment status, contraceptive use and parity are the variables refer to as non-significant in the statement.   Kindly refer to:  **Discussion section, pages 20-21, line 379-381**  for the modifications provided in the revised manuscript   - This has been addressed by replacing the word “Asides” with “Besides” in the revised manuscript for the sake of clarity as pointed out by the reviewer.   Please refer to:  **Discussion section, page 21, line 385** |
| 7. | General comments   - The article with correcting minor comments is publishable. | - We appreciate the comment and recommendation!   Thank you. |
| **II** | **Reviewer #2: Comments** | |
| 1. | 1) Abstract: This is well written what is important is for the authors to indicate the inter cluster correlation coefficient and indicate how it is imperative to delineate the influence across the clusters. | We appreciate the reviewer for this important comment and observation.  The intra cluster correlation coefficient are expressed in percentage as 11.1% (overweight) and 3.0% (underweight) and as such indicated in the abstract. We hope the revised statement suggests how it is imperative to delineate the influence across the clusters.  Please refer to  **Abstract, page 2, lines 44-52**  in the revised version of the manuscript for further detail of corrections and modifications. |
| 2. | 2) The abstract conclusions and recommendation is a bit vague, it is not capturing the key policy statement that the study is advocating o DBM. I suggest revisionso that policy direct is clearly indicated and to the point. | Based on this comment, we have revised and provided statements to capture key policy the study is advocating for effected the changes in the revised version of the manuscript.  Kindly refer to  **Abstract, page 3, lines 47-52**. |
| 3. | Background  3) Line 53- requires updated reference that is recent as 2014 is way too far. I suggest revision of the reference.    Line 54-55 Requires revision as it is grammatically unbalanced. For instance the risk of dying largely occasioned by external causes of death” it is not wellbalance as death is already referenced and this is confusing the readers.  4) Line 59, maternal and child diseases and deaths requires revision.    5) Line 52-60. There was need for the authors to put the argument clears they are discussing unhealth body weight among women and it is not coming out clear even from a world perspective with evidence of the countries that are experiencing this situation which is reading to increased levels of morbidity and mortality among both the vulnerable youth and women. I suggest this must be incorporated.  6) Line 62, before going into Malawi, there is need for the author to justify the statement of SSA by providing a little evidence to justify such statement to highlight the gravity of the problem.    7) Line 65 and 66-The authors need to explain what the population level. The study by Bulirani et al. 2018 looked at the factors affecting nutrition status of reproductive age among women of Dedza District, Malawi was it a multi-level study or not to justify the argument? Please elaborate. | These comments by the reviewer is noted.   - We have revised and updated the reference with an up-to-date reference from WHO fact sheet on manuscript, though the information is still the latest.   Please kindly refer to:  **Introduction section, page 3, line 59**   - We regret the mix-up. We have effected the changes in the revised version of the manuscript as appropriate.   Please refer to:  **Introduction section, pages 3-4, lines 68-70**   - We have revised the phrase to read “maternal and child deaths” in the revised version of the manuscript as appropriate   Kindly refer to:  **Introduction section, page 4, line 71**   - We have provided statements to address this important observation from the reviewer by indicating global prevalence of each of the unhealthy weights in the revised version of the document as suggested   Kindly refer to:  **Introduction section, pages 3-4, lines 59-66** **& 72-73**   - Thank you. Statements have been provided to justify the statement on SSA as advised   Please refer to  **Introduction section, page 4, lines 75-78**   - The comments have been attended to in the revised manuscript by explaining what DBM at population level connotes. We also provide statement to indicate that the study was a single-level cross sectional design   Please refer to  **Introduction section, page 4, lines 78-82 & 84**  for the detailed modification in the revised manuscript |
| 4. | 8) Line 110 and Line 120 there is need to explain the selection criteria as to how the 7231 women were identified. I propose a flow chart be drawn to outline the detail. Please adjust.    9) Line 122 and 128 This is well written and outcome variable well defined. But the emphasis shod be the dependent variable defined in the model. I consider that the one not used in the model should be deleted so that the golden thread is well pronounced.  10) Line 145 there is need to add ethnicity as to how its recoded. Make sure that all the variables used in the model are defined in this section.    11) Line 149 is the use of the chi-square appropriate for means? I propose that the use of the means is not appropriate than ordinary frequencies and percentageand the show the chi-square coefficient and p-Values.    12) Line 183 and 188 please indicate the rationale for applying the nested regression. Otherwise the results of having model 1 is not validated as the outcome variable defining the coefficient in models 2 are congruent. I would propose model 1 de dropped and concentrate at model 2 and 3. If the models 2 and 2 areincluded I can foresee issues of multi-collinearity with the model not converging properly. Please validate and retest the models. This is because the community model is in independent model in this case total disaggregated with the model 2. Remember the unit of analysis is always the individual levels in any multi-level analysis.  13) In the multilevel model, there is need to explain in the methodology how community poverty and education was defined. There is need for clarity. Otherwise, it is a bit confusing to a lay reader. If place of residence is dropped in the model 1, it could fit better as a community variable. | - This very important observation by the reviewer is appreciated. We have provided a flow chart to detail the sample population selection criteria.   Kindly refer to  **Methods section, page 6, lines 135 & 140; Fig 1**   - We have modified the statements to effect the necessary changes as commented by the reviewer.   Kindly refer to  **Methods section, page 7, lines 142-148**   - We sincerely appreciate the reviewer comment in this regard. Region and ethnicity variables original coding in the parent study were retained and were not recoded in the course of the analysis. However, we indicated the three categories of the region of residence but range of the 11 categories of ethnicity was indicated.   Kindly refer to  **Methods section, pages 7-8, lines 166-167**   - We sincere apologise for the mix-up. The statement has been modified in the revised version of the manuscript to effect the reviewer’s comment.   Please refer to:  **Methods section, page 8, line 172**   - We appreciate the reviewer for this important observation. Model-1 has been dropped, model-2 becomes model-1 after reanalysed with exclusion of place of residence, model-3 & model-4 become model-2 & model-3 respectively in the revised version of the manuscript.   Kindly refer to  **Methods section, pages 9-10 & 16-17, lines 206-211 & Table 2**   - The operationally definition of community education read as - proportion of women who have at least secondary level of education in the community; community poverty reads as - proportion of women residing in households below the poverty level, 40% of the wealth index in the community. These are indicated in the revised version of the manuscript.   Also, we have dropped replaced model-1 with model-2 with the exclusion of place of residence (as stated above).  Please refer to:  **Methods, page 7, lines 156-158**  for further modifications provided in the revised manuscript |
| 5. | 14) I would like to propose that the results are presented in a mix bag If we have more than one model thus discussed, there is needs to first use within model analysis-here make sure that all significant findings are flagged. Then, the between model which now compare in relative term, the consistency variable thus causing significant variables worth highlighting. Those are the ones to be appreciated for discussion and they assist in creating a comprehensive golden thread. Try to do a self-validation on this as it will improve readability of the paper. | - As advised, we have rewritten the result section by presenting the measures of association (fixed effects) first and thereafter the random effects (measure of variation)   Please refer to:  **Results section, pages 14-18 , lines 279-323**  for further modifications provided in the revised manuscript |
| 6. | 15) Line 393 and 394. Authors need to revisit the sentence and remove the national income. By nature DHS is a cross sectional data micro-level data set whereas national income is a macro-level data. By data science design, they are different hence the claim not justifiable. Similarly, DHS has dietary data which could be defined either directly or as a proxy and can be used. Hence, this sentence require heavy revision is at all deleted completely as it is countering some important data etiquette.  16) Line 400 and Line 408. There is need to isolate the clearly the variables that are significant when estimating the two dependent variables under discussion.    17) There is need to create an evidence based recommendation and this must be based on the consistency findings of the study. In that tertiary result review,there is need to have have a statement that is to stimulate policies. Like the last sentence in the conclusion section is out of context and require extensive review. | Again, we sincerely appreciate the effort of the reviewer.   - National income has been deleted   However, we retained the nonavailability of dietary intake as as a limitation of the study. This is sequel to the fact that the DHS dietary data are newly included in DHS 8. Kindly take a look at the quoted statement from DHS program blog  “Woman’s Questionnaire now includes **Minimum Dietary Diversity for Women** to assess the micronutrient adequacy of women’s diets and their intake of healthy and unhealthy foods. This fills an important data gap in women’s diet quality. Countries will be able to assess women’s diets at the national and sub-national levels and take appropriate action”(<https://blog.dhsprogram.com/nutrition-data-in-dhs-8/>)  We therefore provided a statement which read thus “(such variables were not available for the present 2015-16 MDHS dataset)” in the revised version of the manuscript  Please refer to:  **Results section, page 22, lines 415-416**   - We have provided statements that isolate significant variables in the revised version of the manuscript.   Kindly refer to  **Conclusion section, page 23, lines 423-426**   - We have also provided statements to address he concern of the reviewer in the revised version of the manuscript.   Kindly refer to  **Conclusion section, page 23, lines 430-432** |
| III | **Additional corrections based on authors’ consideration** | Based on our critical considerations   1. We have reproduced Fig 1 which now becomes Fig 2, to describe women’s first birth interval after HIV diagnosis   Kindly refer to  **Results section, Fig 2** in the revised manuscript  **Thank you.** |
